# Severe disease in children hospitalized with a diagnosis of *Plasmodium vivax* in south-eastern Pakistan

**DOI:** 10.1186/1475-2875-11-144

**Published:** 2012-05-02

**Authors:** Salma Shaikh, Hafeezullah Memon, Bhagchand Iohano, Amna Shaikh, Imran Ahmed, J Kevin Baird

**Affiliations:** 1 Pediatrics Department, Liaquat University of Medical and Health Sciences Jamshoro, Hyderabad, Pakistan; 2 Eijkman-Oxford Clinical Research Unit, Jakarta, Indonesia; 3 Nuffield Department of Medicine, Centre for Tropical Medicine, University of Oxford, Oxford, UK; 4 Eijkman-Oxford Clinical Research Unit, Jalan Diponegoro No.69, Jakarta, 10430, Indonesia

**Keywords:** *Plasmodium vivax*, Severe illness, Clinical presentation, Risk, children, Pakistan, Hospital, *Plasmodium falciparum*

## Abstract

**Background:**

Infection by *Plasmodium vivax* has been considered rarely threatening to life, but recent studies challenge this notion. This study documented the frequency and character of severe illness in paediatric patients admitted to a hospital in south-eastern Pakistan with a laboratory-confirmed diagnosis of vivax malaria.

**Methods:**

An observational study of all 180 paediatric patients admitted with any diagnosis of malaria during 2010 was conducted: 128 *P. vivax*; 48 *Plasmodium falciparum*; and four mixed infections of these species. Patients were classified as having severe illness with any of the following indicators: Glascow coma scale <11; ≥2 convulsions; haemoglobin <5g/dL; thrombocytes <50,000/mL; blood glucose <45mg%; >70 breaths/min; or intravenous anti-malarial therapy. Additionally, 64 patients with a diagnosis of vivax malaria were treated during 2009, and the 21 of these having severe illness were included in analyses of the frequency and character of severe illness with that diagnosis.

**Results:**

During 2010, 39 (31%) or 37 (77%) patients with a diagnosis of *P. vivax* or *P. falciparum* were classified as having severe disease. Including the 2009 records of 64 patients having vivax malaria, a total of 60 (31%) patients with severe illness and a diagnosis of *P. vivax* were available. Altered mental status (Glascow coma scale score <11; or ≥2 convulsions) dominated at 54% of the 83 indicators of severe illness manifest among the patients with vivax malaria, as was true among the 37 children with a diagnosis of falciparum malaria and being severely ill; 58% of the 72 indicators of severe disease documented among them. No statistically significant difference appeared in frequencies of any other severe disease indicators between patients diagnosed with vivax or falciparum malaria. Despite such similarities, a diagnosis of falciparum malaria nonetheless came with 3.8-fold (95% CI = 1.8-8.1) higher risk of presenting with severe illness, and 8.0-fold (95% CI = 2.1-31) greater likelihood of presenting with three or more severe disease indicators. Two patients did not survive hospitalization, one each with a diagnosis of falciparum or vivax malaria.

**Conclusions:**

Vivax malaria caused a substantial burden of potentially life-threatening morbidity on a paediatric ward in a hospital in south-eastern Pakistan.

## Background

In Russell’s 1949 text of malariology [1], S.F. Kitchen wrote, “*As a general rule vivax infections exhibit relatively benign characteristics…instances of death (of otherwise healthy adults) due to this parasite alone must indeed be rare*.” This remained the prevailing view of malariologists over the ensuing decades. Only recently has evidence emerged suggesting that, on the contrary, life-threatening illness with a diagnosis of vivax malaria may often be encountered in many endemic settings [2-6]. Kitchen’s experience with this infection, limited largely to adults in non-endemic settings undergoing therapy for neurosyphilis, likely explains the careful qualification of his expressed view. Endemic settings may create circumstances that exacerbate vivax malaria, and paediatric populations may be particularly likely to encounter those as yet unknown determinants. With almost three billion people living at risk of vivax malaria and at least 100 million clinical attacks annually [7,8], the frequency, character, and risks of life-threatening illness associated with a diagnosis of *Plasmodium vivax* represents a conspicuously important global health issue.

Endemic falciparum and vivax malaria occur in Pakistan [9]. At a hospital in south-eastern Pakistan very ill children with a microscopic diagnosis of *P. vivax* have long been noted [10]. Perhaps like other practitioners in resource-limited settings, other undiagnosed causes of severe illness were often presumed: misdiagnosed or cryptic falciparum malaria, or any number of bacterial or viral agents beyond diagnostic reach in poorly resourced laboratories. However, as reports emerged of severe illness associated with vivax malaria, in 2009 an effort was undertaken to systematically collect clinical and laboratory information from patients referred to paediatric ward with a diagnosis of malaria. These studies aimed to characterize the frequency and character of severe disease with a diagnosis of vivax malaria relative to a diagnosis of *Plasmodium falciparum* in the same population.

## Methods

Liaquat University Hospital (LUH), affiliated with Liaquat University of Medical and Health Sciences, is a 1,600-bed private teaching hospital in Hyderabad, Sindh Province, Pakistan. The LUH is the primary referral hospital for the city of Hyderabad and surrounding rural communities. In 2009 the hospital admitted 2,956 adult patients (at least 17 years of age) and 6,395 paediatric patients. Malaria accounted for 433 (15%) and 148 (2.3%) of adult and paediatric admissions, respectively. Most adults had falciparum malaria (72%), whereas children had approximately equal shares of falciparum (57%) and vivax (43%) malaria.

Two paediatric patient populations composed distinct analytical approaches to the studies undertaken: 1) a retrospective case series of 21 patients diagnosed with *P. vivax* mono-infection and classified as having severe illness (collected from June to October 2009 from among the 64 with vivax malaria evaluated during that period); and 2) observational study of the records of 176 patients diagnosed with either *P. falciparum* or *P. vivax* mono-infections and treated in the paediatric department (April to December 2010). All data were gleaned from records of routine patient care. A protocol describing these studies was reviewed and approved by the ethics review board of Liaquat University of Medical and Health Sciences.

During July and August 2009, blood films from 12 patients diagnosed as having severe illness and *P. vivax*, along with 10 others diagnosed as having severe illness and *P. falciparum*, were evaluated by expert microscopists and by standard nested PCR [11] at the US Naval Medical Research Unit #2 (NAMRU-2) in Jakarta, Indonesia. Blood films from an additional nine patients with a diagnosis of *P. vivax* (July to October 2009) were evaluated by expert microscopy at the Eijkman-Oxford Clinical Research Unit in Indonesia. Two certified (by US NAMRU-2 [12]) expert malaria microscopists blindly read blood films from all 39 patients admitted to the paediatric department with a diagnosis of vivax malaria and having severe illness (April to December 2010).

Patients were classified as having severe disease on the basis of objective clinical or laboratory features as follows: Glascow coma scale (GCS) <11; ≥2 episodes of convulsions; haemoglobin level <5 g/dL; respiratory rate >70 breaths/min; thrombocytes <50,000/mL; blood glucose level <45mg%; or the administration of anti-malarials intravenously. Patients exhibiting none of these features were classified as having uncomplicated malaria. Excepting intravenous antimalarial therapy, these criteria were extracted from the WHO criteria for classifying patients with a diagnosis of falciparum malaria as having severe, complicated, and life-threatening disease [13]. We applied these criteria equally to patients with either falciparum or vivax malaria in order to compare frequencies of these syndromes between species of infection. No studies have validated the syndromes or associated values as risk factors for poor outcomes with vivax malaria. In the instance of intravenous therapy, we applied this physician’s judgement outcome as at least incompatible with a classification of uncomplicated illness.

## Results and discussion

**Figure 1:**
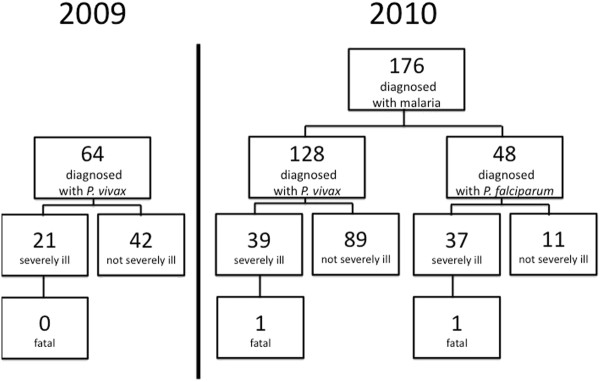
Classification of patients with a diagnosis of malaria treated at the paediatrics ward at Liaquat University Hospital in 2009 and 2010.

During 2009 the paediatric ward admitted 64 children with a diagnosis of vivax malaria (Figure 1). Additional file 1:Table S1 lists clinical and laboratory findings in 21(33%) of those patients classified as having severe illness. The diagnosis of *P. vivax* was confirmed by PCR in 12 of these children, and review by two expert microscopists among all 21. Twelve of those children suffered at least two convulsions (57%) and five had GCS <11 (24%), three of those patients having both. Eight patients (38%) had severe anaemia (<5g/dL), with two of those also having both ≥2 convulsions and GCS < 11. None of the patients exhibited respiratory distress (although most had well above normal respiration rates for their age groups), only one of 12 evaluated had severe thrombocytopaenia (<50,000/mL), another had hypoglycaemia (22mg%), and just one showed a bleed time >8min. All of these children recovered after treatment by either chloroquine syrup or quinine infusion.

Figure 1 illustrates the classification of the 178 patients seen in the paediatric department with a microscopically confirmed diagnosis of falciparum or vivax malaria during 2010. Four other patients had a diagnosis of malaria caused by both *P. falciparum* and *P. vivax*; these patients were excluded from subsequent statistical analyses. Blood films from all of the 39 patients classified as having vivax malaria and severe illness yielded the same diagnosis from the two blinded reads by expert microscopists in Indonesia. Vivax malaria accounted for 71% of malaria cases evaluated, 51% of admissions for malaria, and 51% of cases classified as severely ill. Additional file 2: Table S2 lists clinical and laboratory findings among the 39 patients having at least one severe disease syndrome and a confirmed diagnosis of *P. vivax*. Among the 13 of these managed as outpatients, 12 had severe thrombocytopaenia only, and the other had severe anaemia alone as consistent with the classification as severely ill. Three inpatients also had only severe thrombocytopaenia, but, in contrast to the relatively brief bleed times of those managed as outpatients, they all bled in excess of nine minutes. That observation prompted admission to hospital.

**Figure 2:**
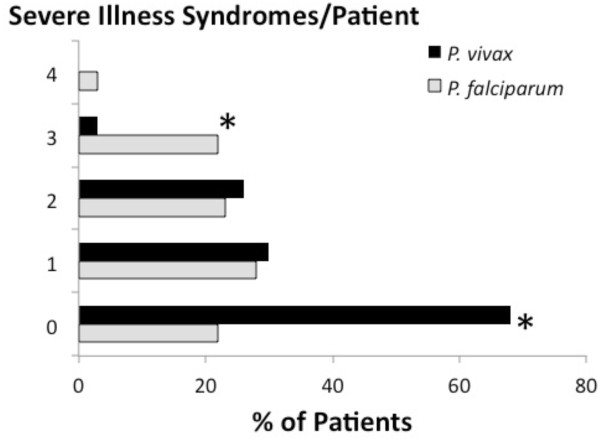
**Distribution of syndromes among patients having at least one qualifying clinical or laboratory feature consistent with a classification of severe malaria with*Plasmodium vivax*(black bars; 83 syndromes among 60 patients) or*Plasmodium falciparum*(grey bars; 72 syndromes among 37 patients).** Comparisons between frequencies for vivax and falciparum malaria within syndromes showed no significant differences (P = 0.171-0.806).

Figure 2 illustrates the distribution of 72 and 83 severe disease syndromes among 37 (2010 only) and 60 (2009 and 2010) patients with falciparum or vivax malaria, respectively. Among comparisons of the distribution frequencies of syndromes between *P. vivax*- and *P. falciparum*-infected patients, no statistically significant differences emerged (P = 0.171-0.802). GCS < 11 and ≥2 convulsions dominated among severe disease syndromes. The proportion of patients classified with either or both of these syndromes was not significantly higher with a diagnosis of *P. falciparum* vs *P. vivax* (OR = 1.7; 95%CI = 0.6-4.7).

**Figure 3:**
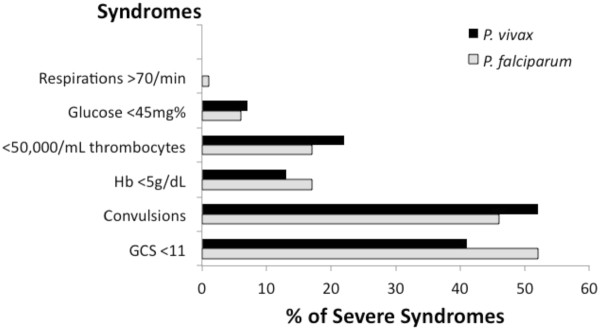
**Relative frequencies of multiple-severe disease syndromes among patients with a diagnosis of*Plasmodium vivax*(black bars; n = 60) or*Plasmodium falciparum. ***(grey bars; n = 37). An asterisk to the right of the bars indicates statistically significant difference. All other comparisons were not significant (P =0.177-0.883).

Figure 3 illustrates the distribution of the number of severe disease syndromes per patient between those diagnosed with *P. vivax* or *P. falciparum*. Vivax malaria came without severe disease much more frequently than falciparum malaria (69% vs 22%; P < 0.001). Likewise, a diagnosis of *P. falciparum* more often came with three or more severe disease syndromes than with *P. vivax* (21% *vs* 3%; P < 0.001). Expressed differently, a diagnosis of falciparum malaria was 3.8 times (95%CI = 1.8-8.1) more likely to be classified as severe (≥1 syndrome), and 8.0 times (95%CI = 2.1-31) more likely to involve more than two indicators of severe illness.

Two deaths attributed to malaria occurred during the study period, one with a diagnosis of *P. falciparum* and one with *P. vivax*. The death with falciparum malaria occurred in a three month-old female admitted with GCS of 6, convulsive, respiratory distress (70b/min), and severe thrombocytopaenia (39,000/mL). She died three days after admission. Death with diagnosis of vivax malaria occurred in a 12 year-old girl admitted with GCS of 10, convulsive, and hypoglycaemic (34mg%). She died seven days after admission.

Other studies in the South Asia region report somewhat similar findings in hospitalized paediatric populations. Among 103 children hospitalized with a diagnosis of vivax malaria at Bikaner, India, 65 (63%) were classified as having severe disease [14]. A similarly high rate of severe illness among the hospitalized in this study occurred: 30/39 (77%). While the Bikaner study found high rates of multi-organ system involvement in vivax malaria (69%), only 19 of the 60 (32%) patients with severe vivax malaria in the current hospital of study in 2009 and 2010 had involvement of more than one organ system. For falciparum malaria in this hospital, 60% of severely ill patients had involvement of more than one severe disease syndrome, much like the 62% rate at Bikaner. Cerebral syndromes in patients with vivax malaria in the current study appeared to be more common, with coma and convulsions accounting for 48% of severely ill patients, compared to just 14% at Bikaner [14]. Severe anaemia appeared among severely ill children with vivax malaria in the current study hospital (18%), but not nearly as frequently as at Bikaner (75%) [14]. Nonetheless, lesser grades of anaemia were certainly a significant problem in the patient population (47% of severely ill with <9g/dL; see Additional files 1: Table S1 and Table S2). Other studies in India [15-18] describe patients having severe vivax malaria characterized by cerebral syndromes, severe thrombocytopaenia, severe anaemia and shock syndromes. Large studies from Indonesia documented severe anaemia, altered mental status, and pulmonary distress among children and adults with a diagnosis of vivax malaria [19,20].

The observational studies described in this report have important limitations. A number of other endemic infections may have affected the patients in this study, but the laboratory capacities required to systemically assess these were lacking. Underlying disease, malnutrition for example, may represent important determinants of exacerbated vivax malaria but none were systematically assessed in these studies. Nonetheless, almost all of the patients in these studies recovered with anti-malarial therapies (few received presumptive antibiotics) and association between the confirmed diagnosis and clinical disease may be considered at least probable. Further prospective studies in endemic zones like south-eastern Pakistan supported by wider arrays of diagnostics for infections and other disease states are needed.

The means of classifying severe illness in this study represents another limitation with these studies. The standard WHO thresholds for disease severity derive from thorough statistical assessments of clinical and laboratory indicators associated with risk of fatal outcome with a diagnosis of falciparum malaria [13]. No such assessments have been carried out with vivax malaria and the current study simply applied those syndromes and thresholds without similar evidence of statistical linkage to a poor prognosis. This approach, uniform disease classification between infecting species, permitted comparison of rates of disease states. The occurrence of coma and convulsions in patients with vivax malaria may be through cellular and molecular processes dissimilar to those thought to occur in falciparum malaria, i.e., by adhesion of infected red blood cells in deep microvasculature. Red cells infected by *P. vivax* show little propensity for such adhesion [21]. Nonetheless, by any direct or indirect mechanism of onset, and regardless of associated risk of death, coma and convulsions may not be reconciled with a classification of uncomplicated illness. The patients with a diagnosis of vivax malaria and classified as severely ill were indeed suffering at least potentially threatening disease states.

## Conclusions

Although a diagnosis of malaria caused by *P. falciparum* carried significantly higher risks of more often severe and more complicated illness, a diagnosis of vivax malaria nonetheless accounted for 51% of the burden of severe illness attributed to malaria. The range and frequency of syndromes – dominated by altered mental status, severe thrombocytopaenia, and severe anaemia – were indistinguishable between patients with either diagnosis. The expressed caveat of Kitchen on the improbability of threatening disease with vivax malaria, i.e., *“…(in otherwise healthy adults)…”,* may prove historically important as better understanding of vivax malaria as it occurs in endemic zones emerges, especially in children. Although relative risks varied between falciparum and vivax malaria, both of these diagnoses came with the same range of potentially life-threatening syndromes at very substantial rates with respect to overall burdens. A diagnosis of infection by *P. vivax*, regardless of what co-factors or determinants may come into play, often came with clinically threatening conditions in the patients evaluated in this study. The long-held view of vivax malaria as a benign infection may threaten patients by inviting inappropriately casual attention to its clinical management, research, and control. The commitment of just 3.1% of global funding for malaria research to *P. vivax* between 2007 and 2009 [22] suggest that such may be the case.

## Abbreviations

LUH: Liaquat University Hospital; NAMRU-2: U.S. Naval Medical Research Unit No.2; GCS: Glascow coma scale; WHO: World Health Organization.

## Competing interests

The authors declare that they have no competing interests.

## Authors’ contributions

SS organized and supervised the collection of data from patient files. HM, AS, IA, and BI identified, collected, entered, and quality-assured the data from patient files. JKB advised on the collection, analysis, and reporting of the data. All authors contributed to writing the manuscript. All authors read and approved the final manuscript.

## Supplementary Material

Additional file 1 **Table S1.** Case series summary of 21 patients classified as having severe disease with a diagnosis of *vivax* malaria during 2009. Click here for file

Additional file 2 **Table S2.** Case series summary of 39 patients classified as having severe disease with a diagnosis of *vivax* malaria during 2010. Click here for file
