# Supplementary material for: Severe disease in children hospitalized with a diagnosis of Plasmodium vivax in south-eastern Pakistan
Source: Malar J. 2012 May 2;11:144. doi: 10.1186/1475-2875-11-144 (PMC3480837; doi:10.1186/1475-2875-11-144)
Supplement: Additional file 1 — Table S1. Case series summary of 21 patients classified as having severe disease with a diagnosis of vivax malaria during 2009. [file 1475-2875-11-144-S1.doc]

**Table S1**. Case series summary of 21 patients classified as having severe disease with a diagnosis of *vivax* malaria during 2009.

| **Patient #** | **Sex** | **Age (yrs)** | **Microscopic Diagnosis** | **PCR Diagnosis** | **GCS*** | **>2 Convul-sions** | **Hb**  **(g/dL)^#^** | **Resp. per min** | **Jaundice** | **Bleed time (mins)** | **Thrombo. X10^3^/mL** | **Blood glucose (mg%)** |
| --- | --- | --- | --- | --- | --- | --- | --- | --- | --- | --- | --- | --- |
| 1 | M | 4 | *P. vivax* | *P. vivax* | 15 | No | 11.6 | 30 | No | 2:40 | 310 | 66 |
| 2 | M | 5 | *P. vivax* | *P. vivax* | 15 | Yes | 11.2 | 32 | No | 4:10 | 79 | 138 |
| 3 | M | 3 | *P. vivax* | *P. vivax* | 15 | Yes | 8.9 | 30 | No | 2:50 | 396 | 59 |
| 4 | F | 2 | *P. vivax* | *P. vivax* | 15 | Yes | 11.5 | 36 | No | 1:40 | 431 | 90 |
| 5 | F | 3 | *P. vivax* | *P. vivax* | 8 | Yes | 9.5 | 20 | No | 3:30 | 69 | 120 |
| 6 | F | 5 | *P. vivax* | *P. vivax* | 4 | No | 11.6 | 40 | No | 2:00 | 430 | 85 |
| 7 | F | 6 | *P. vivax* | *P. vivax* | 4 | Yes | 8.9 | 28 | No | 1:40 | 320 | 70 |
| 8 | F | 10 | *P. vivax* | *P. vivax* | 15 | No | 2.9 | 50 | No | 1:50 | 212 | 80 |
| 9 | M | 10 | *P. vivax* | *P. vivax* | 15 | No | 3.8 | 36 | No | 4:40 | 118 | 83 |
| 10 | F | 3 | *P. vivax* | *P. vivax* | 15 | No | 4.6 | 36 | No | 3:10 | 210 | 115 |
| 11 | F | 11 | *P. vivax* | *P. vivax* | 15 | No | 4.6 | 36 | No | 2:20 | 294 | 90 |
| 12 | M | 5mo | *P. vivax* | *P. vivax* | 15 | No | 4.9 | 50 | No | 6:10 | 35 | 80 |
| 13 | F | 2 | *P. vivax* | ND | 15 | Yes | 9.3 | 40 | No | 4:00 | ND | 61 |
| 14 | F | 7 | *P. vivax* | ND | 4 | Yes | 4.8 | 60 | Yes | 4:20 | ND | 22 |
| 15 | M | 4 | *P. vivax* | ND | 15 | Yes | 4.8 | 28 | No | 6:40 | ND | 104 |
| 16 | M | 7 | *P. vivax* | ND | 8 | Yes | 9.8 | 35 | No | 5:40 | ND | 70 |
| 17 | M | 9 | *P. vivax* | ND | 15 | No | 4.6 | 36 | No | 6:40 | ND | 109 |
| 18 | M | 7mo | *P. vivax* | ND | 15 | Yes | 7.4 | 30 | No | 5:00 | ND | 120 |
| 19 | M | 3 | *P. vivax* | ND | 15 | Yes | 8.6 | 32 | No | 3:40 | ND | 144 |
| 20 | M | 3 | *P. vivax* | ND | 15 | Yes | 7.9 | 35 | No | 5:10 | ND | 80 |
| 21 | F | 12 | *P. vivax* | ND | 10 | No | 9.4 | 30 | No | 8:30 | ND | 90 |
| * Glascow Coma Scale; # Haemoglobin concentration; ^ Respirations/minute | | | | | | | | | | | | |
