# Supplementary material for: Severe disease in children hospitalized with a diagnosis of Plasmodium vivax in south-eastern Pakistan
Source: Malar J. 2012 May 2;11:144. doi: 10.1186/1475-2875-11-144 (PMC3480837; doi:10.1186/1475-2875-11-144)
Supplement: Additional file 2 — Table S2. Case series summary of 39 patients classified as having severe disease with a diagnosis of vivax malaria during 2010. [file 1475-2875-11-144-S2.doc]

**Table S2**. Case series summary of 39 patients classified as having severe disease with a diagnosis of *vivax* malaria during 2010.

| **Patient #** | **Sex** | **Age (yrs)** | **GCS*** | **>2 Convulsions** | **Hb (g/dL)^#^** | **Resp.^ per min** | **Bleed time (min)** | **Thrombo.X10^3^/mL~** | **Blood glucose (mg%)** | **Admitted** | **Intravenous Quinine or Artesunate** |
| --- | --- | --- | --- | --- | --- | --- | --- | --- | --- | --- | --- |
| 1 | M | 3 | 15 | No | 5.4 | 30 | 4:30 | 116 | 94 | Yes | Yes |
| 2 | F | 9 | 15 | No | 9.7 | 36 | 6:00 | 46 | 78 | Yes | No |
| 3 | M | 1 | 15 | No | 5.1 | 32 | 2:05 | 224 | 76 | Yes | Yes |
| 4 | F | 5 | 15 | No | 3.1 | 36 | 5:10 | 100 | 64 | Yes | Yes |
| 5 | F | 7 | 8 | Yes | 10.4 | 32 | 6:30 | 282 | 90 | Yes | Yes |
| 6 | F | 7 | 13 | Yes | 9.9 | 30 | 5:20 | 187 | 47 | Yes | Yes |
| 7 | M | 6 | 10 | Yes | 8.6 | 26 | 9:10 | 35 | 66 | Yes | Yes |
| 8 | F | 2 | 15 | No | 10.6 | 26 | 6:10 | 49 | 35 | Yes | Yes |
| 9 | M | 10 | 15 | No | 4.9 | 26 | 3:10 | 140 | 90 | No | No |
| 10 | F | 2 | 15 | No | 7.8 | 34 | 2:10 | 45 | 88 | No | No |
| 11 | M | 10 | 15 | No | 12.2 | 30 | 2:40 | 49 | 75 | No | No |
| 12 | M | 5 | 15 | No | 9.3 | 31 | 1:40 | 49 | 90 | No | No |
| 13 | M | 11 | 15 | No | 11.5 | 25 | 3:20 | 47 | 105 | No | No |
| 14 | M | 5 | 15 | No | 11.8 | 40 | 9:40 | 25 | 88 | Yes | Yes |
| 15 | F | 12 | 15 | No | 11.3 | 27 | 3:00 | 49 | 32 | Yes | No |
| 16 | F | 1 | 15 | No | 6.6 | 40 | 3:20 | 47 | 78 | No | No |
| 17 | M | 5 | 8 | Yes | 11.5 | 40 | 3:20 | 370 | 88 | Yes | Yes |
| 18 | M | 1 | 8 | Yes | 11.3 | 34 | 4:30 | 125 | 82 | Yes | Yes |
| 19 | F | 3 | 15 | No | 9.6 | 42 | 4:00 | 47 | 70 | No | No |
| 20 | M | 1 | 15 | No | 6.6 | 48 | 9:00 | 27 | 50 | Yes | Yes |
| 21 | M | 3 | 15 | No | 8.5 | 30 | 3:00 | 40 | 102 | No | No |
| 22 | F | 1 | 15 | No | 6.8 | 48 | 2:40 | 46 | 66 | No | No |
| 23 | M | 6 | 10 | Yes | 12.8 | 25 | 2:20 | 195 | 72 | Yes | Yes |
| 24 | M | 10 | 15 | No | 11.2 | 28 | 4:20 | 44 | 74 | No | No |
| 25 | M | 3 | 15 | Yes | 21.3 | 30 | 3:00 | 145 | 74 | Yes | No |
| 26 | M | 5 | 15 | No | 9.1 | 27 | 3:40 | 78 | 38 | Yes | Yes |
| 27 | M | 10 | 15 | No | 12.2 | 30 | 4:20 | 50 | 80 | No | No |
| 28 | M | 11 | 8 | Yes | 12.8 | 36 | 3:40 | 157 | 86 | Yes | Yes |
| 29 | F | 3 | 15 | No | 5.2 | 38 | 3:55 | 49 | 84 | No | No |
| 30 | F | 10 | 15 | No | 5.1 | 24 | 4:20 | 47 | 100 | No | No |
| 31 | F | 4 | 15 | No | 5.8 | 34 | 3:40 | 80 | 40 | Yes | Yes |
| 32 | F | 5 | 10 | Yes | 11.5 | 36 | 1:40 | 262 | 70 | Yes | Yes |
| 33 | F | 5 | 10 | Yes | 10.7 | 46 | 4:20 | 88 | 68 | Yes | Yes |
| 34 | M | 3 | 8 | Yes | 9.4 | 40 | 2:40 | 292 | 74 | Yes | Yes |
| 35 | M | 6 | 8 | Yes | 10.3 | 26 | 3:55 | 87 | 70 | Yes | Yes |
| 36 | F | 5 | 15 | No | 4.5 | 64 | 2:50 | 78 | 70 | Yes | Yes |
| 37 | M | 8 | 15 | Yes | 11.8 | 20 | 2:10 | 141 | 72 | Yes | Yes |
| 38 | F | 3 | 15 | Yes | 11.4 | 34 | 2:05 | 175 | 65 | Yes | Yes |
| 39 | F | 12 | 10 | Yes | 8.7 | 36 | 3:55 | 94 | 34 | Yes | Yes^ϕ^ |
| * Glascow Coma Scale; # Haemoglobin concentration; ^ Respirations/minute; ϕ Patient not surviving | | | | | | | | | | | |
